# Amyloid pathology modulates the relationship between subsyndromal symptomatic depression and tau accumulation in non-demented older adults

**DOI:** 10.3389/fnagi.2025.1679285

**Published:** 2025-12-10

**Authors:** Jiahe Bai, Xiaonan Wei, Hongchun Wei, Yaojun Tai, Min Kong, Maowen Ba, Chunhua Zhang

**Affiliations:** 1 Heze Hospital Affiliated of Shandong First Medical University, Heze, China; 2 Shandong First Medical University and Shandong Academy of Medical Sciences, Jinan, China; 3 Department of Neurology, Affiliated Yantai Yuhuangding Hospital of Qingdao University, Yantai, China; 4 Shandong Provincial Key Laboratory of Neuroimmune Interaction and Regulation, Yantai, China; 5 Department of Neurology, Jiaozhou Branch of Shanghai East Hospital, Tongji University, Jiaozhou, China; 6 Department of Neurology, Yantaishan Hospital, Yantai, China

**Keywords:** subsyndromal symptomatic depression, amyloid-β, tau, Alzheimer’s disease, neuroimaging biomarkers

## Abstract

**Objective:**

Subsyndromal symptomatic depression (SSD) has been increasingly implicated in the pathophysiological processes of Alzheimer’s disease (AD). However, it remains unclear whether SSD and amyloid-β (Aβ) pathology jointly contribute to tau deposition. This study aimed to investigate the interaction between SSD and Aβ status on regional tau accumulation in non-demented older adults.

**Materials and methods:**

We analyzed data from 391 non-demented older adults in the Alzheimer’s Disease Neuroimaging Initiative (ADNI) who underwent Aβ and tau positron emission tomography (PET) scans, as well as Geriatric Depression Scale (GDS-15) assessments. Aβ positivity (Aβ+) was defined by established tracer-specific standardized uptake value ratio (SUVR) thresholds (≥1.11 for ^18^F-florbetapir or ≥1.08 for ^18^F-florbetaben). SSD was defined as a GDS-15 score of 1–5. Linear mixed-effects models were applied to assess the longitudinal effects of SSD and Aβ status on regional tau accumulation over 2 years.

**Results:**

At baseline, significant interactions between SSD and Aβ status were observed for regional tau SUVRs, with the Aβ+/SSD+ group exhibiting significantly higher tau levels across all Braak stages compared with the other groups. Longitudinal analyses identified a significant three-way interaction among SSD, Aβ status, and time in the Braak III/IV and Braak V/VI regions. Moreover, the Aβ+/SSD+ group demonstrated significantly faster tau accumulation compared to all other groups. The Aβ+/SSD− group also exhibited greater tau accumulation than the Aβ−/SSD− group, whereas no significant differences were observed between the Aβ− groups.

**Conclusion:**

These findings suggest that SSD is associated with greater early tau accumulation in individuals with Aβ pathology.

## 1 Introduction

Dementia represents a major and growing global health concern, with rising prevalence and significant social and economic impacts. By 2050, the number of people living with dementia is expected to more than double, placing unprecedented strain on healthcare systems worldwide (GBD 2019 Dementia Forecasting Collaborators, 2022). Late-life depression is common in older adults and has consistently been associated with an increased risk of developing dementia, including Alzheimer’s disease (AD) (Green et al., 2003; Diniz et al., 2013; Bennett and Thomas, 2014; Ly et al., 2021). Meta-analyses further indicate that individuals with depression have approximately twice the risk of developing dementia compared to those without depressive symptoms (Jorm, 2001; Ownby et al., 2006).

Depressive symptoms range from mild subthreshold conditions, such as subsyndromal symptomatic depression (SSD), to major depressive disorder (MDD). SSD is more prevalent than MDD in older adults and has been associated with functional disability (Hybels et al., 2009) and cognitive impairment (Boyle et al., 2010). Longitudinal studies have shown that SSD is linked to an approximately threefold higher risk of developing dementia in non-demented older adults (Oh et al., 2021), suggesting that SSD may represent an early and potentially modifiable marker of dementia risk. Recent meta-analytic evidence has identified depression, along with age, APOE ε4, and lower education, as key predictors of cognitive decline among non-demented individuals (Li et al., 2023). Neuroimaging studies have consistently associated SSD with accelerated cognitive decline and regional brain atrophy, particularly in AD-vulnerable regions such as the hippocampus and temporal cortex (Zhang et al., 2020; Jing et al., 2024). Importantly, emerging evidence suggests that SSD is also associated with AD-related pathology. SSD has been shown to exacerbate cognitive deterioration in the presence of elevated amyloid-β (Aβ) burden (Zhang et al., 2020), suggesting a potential interaction between depressive symptoms and amyloid pathology. Similarly, recent evidence has shown that individuals with both Aβ positivity and mild depressive symptoms have the fastest structural brain atrophy and metabolic decline, along with more rapid cognitive deterioration (Zhang et al., 2025). Beyond these Aβ-related effects, cross-sectional neuroimaging studies have also demonstrated higher tau burden in individuals with depressive symptoms (Gatchel et al., 2017), raising the possibility that depression may be linked not only to Aβ burden but also to tau pathology. However, it remains unclear whether SSD and Aβ pathology jointly contribute to tau accumulation.

To address this gap, the present study investigated whether amyloid pathology modulates the association between SSD and regional tau accumulation in non-demented older adults. We hypothesized that SSD would be associated with greater tau accumulation primarily in the presence of Aβ positivity.

## 2 Materials and methods

### 2.1 Data sources

Participants for this study were drawn from the Alzheimer’s Disease Neuroimaging Initiative (ADNI) database,^1^ with data downloaded on October 10, 2023. ADNI was launched in 2003 under the leadership of Dr. Michael W. Weiner. It incorporates serial MRI, positron emission tomography (PET), fluid biomarker measurements, and comprehensive neuropsychological assessments to support the early detection and longitudinal tracking of AD.

### 2.2 Participants

A total of 391 non-demented participants were included from the ADNI database. Eligibility required availability of a baseline amyloid PET scan (^18^F-florbetapir or ^18^F-florbetaben) and longitudinal ^18^F-flortaucipir tau PET imaging. All baseline scans were acquired within a six-month window. Participants were classified as cognitively normal (CN; MMSE > 24, CDR = 0) or as having mild cognitive impairment (MCI; MMSE > 24, CDR = 0.5, objective memory impairment on the education-adjusted Wechsler Memory Scale II, preserved activities of daily living) according to ADNI diagnostic protocols. Detailed inclusion and exclusion criteria are available on the ADNI website (see text footnote 1).

### 2.3 Standard protocol approvals, registrations, and patient consents

The ADNI study was approved by the Institutional Review Board of each participating site, and written informed consent was obtained from all participants. The present analyses used data from the ADNI-3 phase, which is registered at ClinicalTrials.gov (NCT02854033).

### 2.4 Depression scale measurement

Depressive symptoms were assessed using the 15-item Geriatric Depression Scale (GDS-15) in the ADNI cohort. Total scores range from 0 to 15, with higher scores indicating greater severity of depressive symptoms. A GDS-15 score of ≥ 6 is generally considered indicative of clinically significant depression (Marc et al., 2008). Consistent with prior studies (Mackin et al., 2012; Bertens et al., 2017; Zhang et al., 2020), SSD was defined as a GDS-15 score of 1–5 (coded as SSD+), whereas a score of 0 indicated the absence of depressive symptoms (coded as SSD−).

### 2.5 PET imaging biomarkers

Amyloid PET scans were acquired 50–70 min after intravenous injection of ^18^F-florbetapir or 90–110 min after injection of ^18^F-florbetaben, each scan was reconstructed into 4 × 5-min frames. Tau PET imaging was performed 75–105 min after injection of ^18^F-flortaucipir and reconstructed into 6 × 5-min frames. Detailed information on PET acquisition and preprocessing procedures in ADNI is available at https://adni.loni.usc.edu/data-samples/adni-data/neuroimaging/pet/.

For amyloid PET, standardized uptake value ratios (SUVRs) were calculated by dividing the mean tracer uptake within a predefined cortical composite region comprising the frontal, lateral parietal, anterior and posterior cingulate, and lateral temporal cortices by the uptake in the whole cerebellum (Landau et al., 2012). Aβ positivity (Aβ+) was determined using global SUVR thresholds of ≥1.11 for florbetapir and ≥1.08 for florbetaben (Royse et al., 2021). Amyloid PET data were obtained from the ADNI files: “UCBERKELEYAV45_04_26_22.csv” for florbetapir and “UCBERKELEYFBB_04_26_22.csv” for florbetaben.

For tau PET, SUVRs were calculated for three composite regions of interest (ROIs) approximating Braak stages I, III/IV, and V/VI. Braak stage I included the entorhinal cortex. Braak stages III/IV included the parahippocampal gyri, fusiform gyri, lingual gyri, amygdala, middle temporal gyri, inferior temporal gyri, insula, anterior cingulate cortex, posterior cingulate cortex, isthmus cingulate cortex, and temporal poles. Braak stages V/VI included the frontal poles, superior frontal gyri, middle frontal gyri, lateral orbitofrontal gyri, medial orbitofrontal gyri, pars opercularis, pars orbitalis, pars triangularis, supramarginal gyri, superior parietal lobules, inferior parietal lobules, lateral occipital cortex, precuneus, banks of the superior temporal sulcus, superior temporal gyri, and transverse temporal gyri. In addition, a meta-temporal region was examined, defined as the bilateral entorhinal cortex, amygdala, fusiform gyrus, and inferior and middle temporal cortices, based on FreeSurfer segmentation as specified in the “Meta Temporal ROI” section (Jack et al., 2017). The Braak stage II region (hippocampus) was excluded from all analyses due to potential off-target binding from the adjacent choroid plexus (Lemoine et al., 2018). Longitudinal tau PET data were acquired at baseline, 1-year, and 2-year follow-up visits using 18F-flortaucipir and retrieved from the ADNI file “UCBERKELEYAV1451_04_26_22.csv.”

### 2.6 Covariate data collection

All covariate information used for statistical adjustment, including age (years, continuous), sex (0 = male, 1 = female), years of education (continuous), APOE ε4 carrier status (0 = non-carrier, 1 = carrier), and diagnostic status (0 = CN, 1 = MCI), was extracted from the ADNI files (“ADNIMERGE.csv” and “APOERES.csv”). These variables were included to control for demographic and genetic factors known to influence both depressive symptoms and AD pathology.

### 2.7 Statistical analysis

All statistical analyses were performed using IBM SPSS Statistics version 26 and R software (version 4.3.3; R Foundation for Statistical Computing, Vienna, Austria). A two-tailed *p* value < 0.05 was considered statistically significant. For continuous variables with a normal distribution, one-way analysis of variance (ANOVA) followed by false discovery rate (FDR) correction was applied to adjust for multiple comparisons. For categorical variables, group differences were assessed using the chi-square test. For non-normally distributed continuous variables, the Kruskal–Wallis test with FDR correction was used.

Regional tau SUVRs were mildly right-skewed. We assessed the normality of model residuals by inspecting residual-versus-fitted plots and normal Q–Q plots and found them to be approximately symmetric and centered around zero, with only modest departures in the tails. Therefore, untransformed tau PET SUVR values were used in all baseline and longitudinal regression models. For baseline analyses, multiple linear regression models were fitted for each tau PET region of interest (ROI). Each model included SSD status, Aβ status, and their interaction term (SSD × Aβ) as predictors, with age, sex, years of education, diagnostic status, and APOE ε4 carrier status as covariates. Multicollinearity among covariates was assessed using variance inflation factors (VIFs), all of which were below 5, indicating no significant collinearity.

For longitudinal analyses, linear mixed-effects models were employed to examine the effects of SSD and Aβ status on changes in regional tau PET SUVRs over time. Each model included the main effects of baseline age, sex, APOE ε4 carrier status, diagnostic status, and years of education, their interactions with time, and a random intercept for each participant. *Post hoc* pairwise comparisons were conducted to evaluate differences in tau accumulation trajectories among the four joint SSD/Aβ groups (Aβ−/SSD−, Aβ−/SSD+, Aβ+/SSD−, and Aβ+/SSD+), with FDR correction applied for multiple comparisons.

## 3 Results

### 3.1 Baseline demographics and clinical characteristics

This study included 391 non-demented participants, comprising 115 Aβ−/SSD−, 124 Aβ−/SSD+, 70 Aβ+/SSD−, and 82 Aβ+/SSD+ individuals (Table 1). Regarding demographic characteristics, the Aβ+/SSD+ group was significantly older and had a higher proportion of APOE ε4 carriers compared with both the Aβ−/SSD− and Aβ−/SSD+ groups. The Aβ+/SSD+ group also showed the highest prevalence of MCI compared with the other three groups. There were no significant differences in sex distribution or years of education among the four groups.

**Table 1:** Baseline characteristics across Aβ/SSD groups in non-demented participants.

Characteristic	Aβ-/SSD-	Aβ-/SSD+	Aβ+/SSD-	Aβ+/SSD+	*P*
	(*N* = 115)	(*N* = 124)	(*N* = 70)	(*N* = 82)	
CN/MCI	100/15 (87%/13%)	74/50 (60%/40%)^*^	51/19 (73%/27%)^*^	33/49 (40%/60%)^*,†,‡^	**<0.001**
Age	70.12 ± 5.62	69.91 ± 7.42	72.40 ± 6.42^*,†^	72.50 ± 7.37^*,†^	**0.004**
Female, *n* (%)	68 (59%)	69 (56%)	37 (53%)	48 (59%)	0.83
Years of education	16.92 ± 2.18	16.44 ± 2.43	16.46 ± 2.10	16.57 ± 2.43	0.4
APOE ε4 carrier, *n* (%)	28 (24%)	30 (24%)	47 (67%)^*,†^	47 (57%) ^*,†^	**<0.001**
Braak I SUVR	1.08 (0.14)	1.07 (0.14)	1.14 (0.29) ^*,†^	1.29 (0.32)^*,†,‡^	**<0.001**
Braak III/IV SUVR	1.09 (0.10)	1.09 (0.09)	1.15 (0.15) ^*,†^	1.20 (0.22)^*,†,‡^	**<0.001**
Braak V/VI SUVR	1.02 (0.09)	1.02 (0.07)	1.07 (0.10) ^*,†^	1.10 (0.15)^*,†,‡^	**<0.001**
Meta Temporal ROI SUVR	1.13 (0.09)	1.13 (0.09)	1.20 (0.16) ^*,†^	1.27 (0.26)^*,†,‡^	**<0.001**

Data are expressed as mean ± SD, median (IQR), or n (%). Aβ–, amyloid-β-negative; Aβ+, amyloid-β-positive; APOE, apolipoprotein E; CN, cognitively normal; MCI, mild cognitive impairment; SSD, Subsyndromal Symptomatic Depression; SSD+, presence of subsyndromal depressive symptoms, defined as a Geriatric Depression Scale (GDS) score of 1–5; SSD−, absence of depressive symptoms (GDS = 0); ROI, Region of Interest; SUVR, Standardized Uptake Value Ratio; *p* values in bold indicate statistical significance (*p* < 0.05). * Significant represented the difference between Aβ**-**/SSD**-** and other groups. † Significant represented the difference between Aβ**-**/SSD+ and Aβ+/SSD**-** or Aβ+/SSD+. ‡ Significant represented the difference between Aβ+/SSD**-** and Aβ+/SSD+.

Regarding regional tau burden, significant group differences were observed across all Braak regions and the meta-temporal region (all *p* < 0.001). The Aβ+/SSD+ group exhibited the highest SUVRs in Braak I, Braak III/IV, Braak V/VI, and the meta-temporal region compared with other groups. The Aβ+/SSD− group also showed significantly higher tau SUVRs than both Aβ− groups, whereas no significant differences were observed between the Aβ−/SSD− and Aβ−/SSD+ groups.

### 3.2 Baseline regional tau differences by SSD and Aβ status

To assess the interaction between Aβ status and SSD on baseline regional tau deposition, multiple linear regression models were fitted for each tau PET ROI (Braak I, Braak III/IV, Braak V/VI, and the meta-temporal region), adjusting for age, sex, education, diagnostic status (CN vs. MCI), and APOE ε4 carrier status. A significant interaction between SSD and Aβ status was observed across all regions, including Braak I (β = 0.65, *p* < 0.001), Braak III/IV (β = 0.50, *p* = 0.007), Braak V/VI (β = 0.46, *p* = 0.015), and the meta-temporal region (β = 0.51, *p* = 0.006), as shown in Table 2.

**Table 2:** Associations of Aβ status, SSD, and their interaction with baseline regional tau SUVRs among non-demented participants.

Term	Braak I SUVR			Braak III/IV SUVR			Braak V/VI SUVR			Meta Temporal ROI SUVR		
	β	95% CI	*P*	β	95% CI	*P*	β	95% CI	*P*	β	95% CI	*P*
APOE ε4	0.26	0.07 ~ 0.45	**0.007**	0.17	−0.03 ~ 0.37	0.093	0.09	−0.11 ~ 0.29	0.392	0.18	−0.02 ~ 0.38	0.077
Age	−0.02	−0.11 ~ 0.07	0.623	−0.04	−0.13 ~ 0.06	0.425	−0.14	−0.24 ~ −0.04	0.005	−0.01	−0.11 ~ 0.08	0.769
Gender	0.12	−0.06 ~ 0.29	0.193	0.15	−0.04 ~ 0.33	0.124	0.27	0.08 ~ 0.46	0.005	0.15	−0.04 ~ 0.33	0.125
Education	0.09	0.00 ~ 0.17	0.043	0.04	−0.05 ~ 0.13	0.384	0.07	−0.02 ~ 0.16	0.14	0.02	−0.07 ~ 0.11	0.669
Diagnosis	0.28	0.19 ~ 0.37	<0.001	0.23	0.13 ~ 0.32	<0.001	0.17	0.07 ~ 0.27	0.001	0.24	0.14 ~ 0.34	<0.001
SSD	−0.23	−0.45 ~ −0.01	0.039	−0.19	−0.42 ~ 0.05	0.118	−0.12	−0.35 ~ 0.12	0.323	−0.18	−0.41 ~ 0.05	0.134
Aβ	0.38	0.11 ~ 0.64	0.006	0.41	0.13 ~ 0.70	0.004	0.45	0.16 ~ 0.74	0.002	0.41	0.13 ~ 0.69	0.005
SSD+ × Aβ+	0.65	0.31 ~ 0.99	**<0.001**	0.5	0.14 ~ 0.87	**0.007**	0.46	0.09 ~ 0.83	**0.015**	0.51	0.15 ~ 0.87	**0.006**

Aβ+, amyloid-β-positive; APOE, apolipoprotein E; SSD, subsyndromal symptomatic depression; SSD+, presence of subsyndromal depressive symptoms, defined as a Geriatric Depression Scale (GDS) score of 1–5; SUVR, standardized uptake value ratio; CI, confidence interval. Linear regression models were adjusted for age, gender, education, diagnostic status (CN vs. MCI), and APOE ε4 status. β values represent the estimated effect size for each predictor on regional tau SUVRs. *p* values in bold indicate statistical significance (*p* < 0.05).

### 3.3 Longitudinal change models

To examine longitudinal changes in tau accumulation, linear mixed-effects models were used to assess the effects of Aβ status, SSD, and their interaction with time on regional tau PET SUVRs over a two-year follow-up period.

Significant three-way interactions among Aβ status, SSD, and time were observed in the Braak III/IV (estimate = 0.0335, *p* = 0.049) and Braak V/VI (estimate = 0.0285, *p* = 0.047; see Table 3) regions, indicating that the joint presence of Aβ pathology and SSD was associated with a faster rate of tau accumulation over time.

**Table 3:** Longitudinal linear mixed-effects models for changes in regional tau PET SUVRs among non-demented participants.

Term	Braak I SUVR			Braak III/IV SUVR			Braak V/VI SUVR			Meta temporal ROI SUVR		
	Estimate	SE	*P*	Estimate	SE	*P*	Estimate	SE	*P*	Estimate	SE	*P*
Age X time	0.0008	0.0008	0.3586	−0.0002	0.0006	0.7763	−0.0001	0.0005	0.8116	−0.0002	0.0008	0.7600
APOE ε4 X time	0.0001	0.0113	0.9951	0.0080	0.0087	0.3612	0.0077	0.0073	0.2933	0.0110	0.0104	0.2913
Diagnosis X time	−0.0020	0.0115	0.8648	0.0180	0.0088	0.0425	0.0175	0.0074	0.0200	0.0227	0.0105	0.0317
Education X time	0.0028	0.0025	0.2525	−0.0002	0.0019	0.9024	−0.0007	0.0016	0.6403	−0.0008	0.0023	0.7306
Gender X time	0.0114	0.0114	0.3180	−0.0017	0.0088	0.8458	0.0025	0.0074	0.7375	−0.0018	0.0104	0.8644
Aβ X time	0.0029	0.0187	0.8779	0.0047	0.0144	0.7452	−0.0021	0.0121	0.8609	0.0104	0.0171	0.5455
SSD X time	−0.0179	0.0175	0.3061	−0.0251	0.0134	0.0633	−0.0236	0.0113	0.0387	−0.0227	0.0160	0.1570
Aβ X SSD X time	0.0358	0.0220	0.1056	0.0335	0.0169	**0.0493**	0.0285	0.0142	**0.0473**	0.0333	0.0201	0.1002

Aβ, amyloid-β; SSD, Subsyndromal Symptomatic Depression; APOE, apolipoprotein E; SUVR, standardized uptake value ratio; Linear mixed models were used to evaluate longitudinal changes in tau PET SUVRs across regions of interest (Braak I, Braak III/IV, Braak V/VI, and meta-temporal). First, changes related to Aβ status and SSD were assessed in the same model. Then, an interaction between Aβ status and SSD was added. Main effects of independent variables are included in each model (estimates not shown). Estimates are unstandardized values, reflecting the amount of change in each dependent variable per year. *p* values in bold indicate statistical significance (*p* < 0.05).

**Figure 1:**
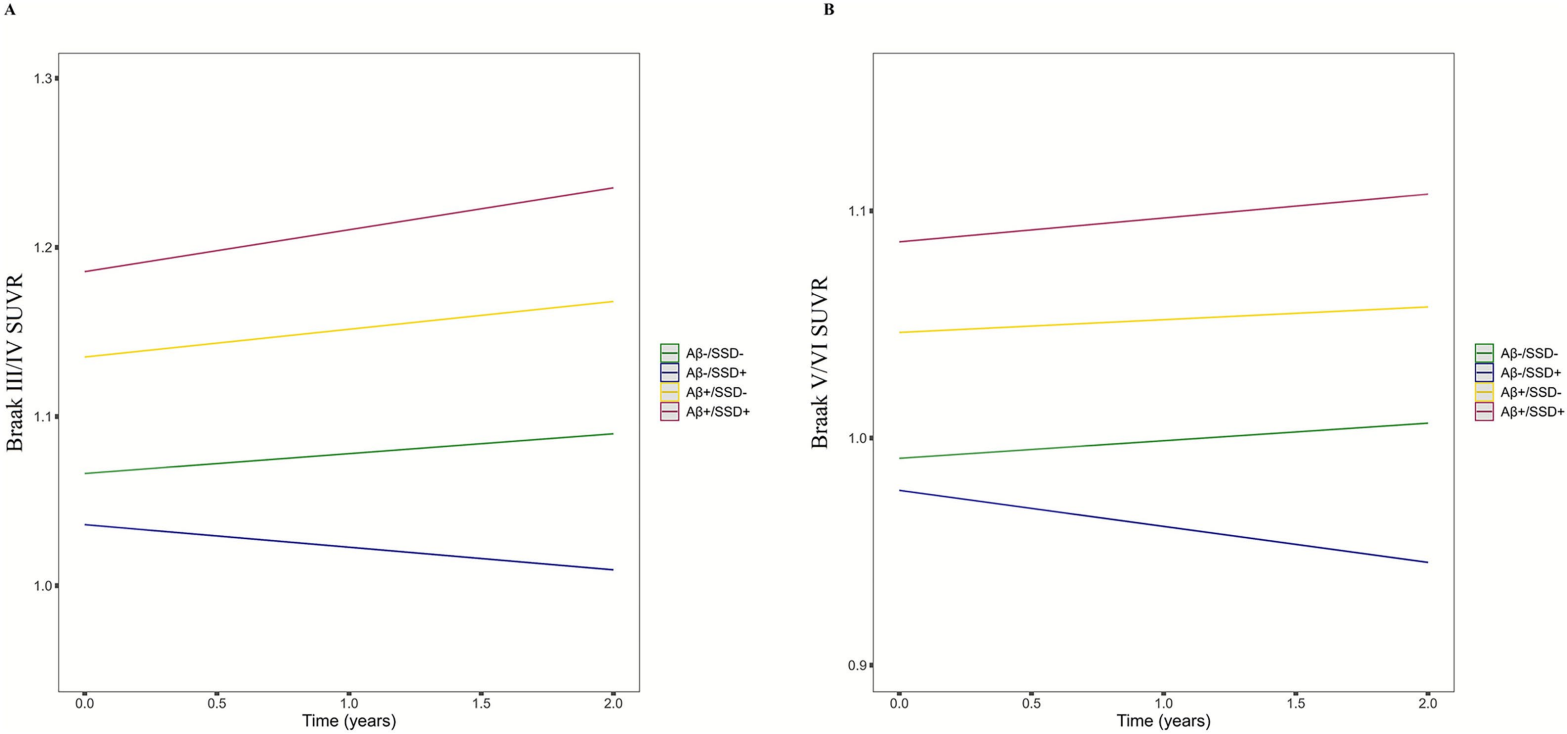
Longitudinal changes in tau accumulation by joint A*β* and SSD status. Aβ−, amyloid-β-negative; Aβ+, amyloid-β-positive; SSD, subsyndromal symptomatic depression; SSD+, presence of SSD (GDS score 1–5); SSD−, absence of depressive symptoms (GDS = 0). The Aβ+/SSD + group exhibited higher tau accumulation over time compared with the other groups in the Braak III/IV (A) and Braak V/VI (B) regions.

To further characterize these effects, *post hoc* pairwise comparisons were performed across the four groups (Aβ−/SSD−, Aβ−/SSD+, Aβ+/SSD−, and Aβ+/SSD+), as shown in Table 4 and Figures 1A,B. The Aβ+/SSD+ group exhibited significantly greater annual tau SUVRs in Braak III/IV (*p* = 0.043) and Braak V/VI (*p* = 0.035) compared with the Aβ+/SSD− group. Moreover, relative to both Aβ− groups, the Aβ+/SSD+ group showed markedly faster tau accumulation in Braak III/IV and Braak V/VI (all *p* < 0.001). Consistent with established Aβ-related tau propagation patterns, the Aβ+/SSD− group exhibited significantly faster tau accumulation in Braak III/IV (*p* = 0.007) and Braak V/VI (*p* = 0.005) compared with the Aβ−/SSD− group. However, no significant differences were observed between the Aβ−/SSD− and Aβ−/SSD+ groups.

**Table 4:** Pairwise comparisons of longitudinal tau accumulation across Aβ/SSD groups among non-demented participants.

Contrast	Braak III/IV SUVR			Braak V/VI SUVR		
	Estimate	SE	*P*	Estimate	SE	*P*
(Aβ−/SSD− X time) vs. (Aβ−/SSD+ X time)	0.039	0.021	0.056	0.023	0.015	0.144
(Aβ−/SSD− X time) vs. (Aβ+/SSD− X time)	−0.071	0.025	**0.007**	−0.055	0.019	**0.005**
(Aβ−/SSD− X time) vs. (Aβ+/SSD+ X time)	−0.124	0.024	**<0.001**	−0.096	0.018	**<0.001**
(Aβ−/SSD+ X time) vs. (Aβ+/SSD− X time)	−0.110	0.024	**<0.001**	−0.077	0.018	**<0.001**
(Aβ−/SSD+ X time) vs. (Aβ+/SSD+ X time)	−0.163	0.023	**<0.001**	−0.119	0.017	**<0.001**
(Aβ+/SSD− X time) vs. (Aβ+/SSD+ X time)	−0.054	0.025	**0.043**	−0.042	0.019	**0.035**

Aβ−, amyloid-β negative; Aβ+, amyloid-β positive; SSD, subsyndromal symptomatic depression; SSD+, presence of subsyndromal depressive symptoms, defined as a Geriatric Depression Scale (GDS) score of 1–5; SSD−, absence of depressive symptoms (GDS = 0); SUVR, standardized uptake value ratio. Estimates represent unstandardized beta coefficients derived from linear mixed-effects models, indicating the rate of annual change in tau PET SUVRs. *p* values in bold indicate statistical significance after FDR correction (adjusted *p* < 0.05).

### 3.4 Sensitivity analysis

To assess the robustness of our findings, we repeated the baseline and longitudinal analyses using square-root–transformed tau-PET SUVRs to address potential non-normality. At baseline, the interaction between SSD and Aβ status remained significant across all examined regions (Supplementary Table S1). In the longitudinal models, a significant three-way interaction among Aβ status, SSD, and time was observed in the Braak V/VI region (estimate = 0.013, *p* = 0.046; Supplementary Table S2). The corresponding interaction in Braak III/IV showed a similar pattern but did not reach statistical significance. Pairwise comparisons further showed that the Aβ+/SSD+ group exhibited the greatest annual increase in tau-PET SUVRs in both Braak III/IV and Braak V/VI compared with other groups (Supplementary Table S3). These findings indicate that the pattern of associations was consistent after transformation.

## 4 Discussion

The present study identified significant interactions between SSD and Aβ pathology in relation to regional tau burden among non-demented older adults. At baseline, significant interactions between SSD and Aβ status were observed for regional tau SUVRs, with the Aβ+/SSD+ group exhibiting significantly higher tau accumulation across all Braak stages compared with the other groups. Longitudinal analyses further identified a significant three-way interaction among SSD, Aβ status, and time in the Braak III/IV and Braak V/VI regions, suggesting that the joint presence of SSD and Aβ pathology accelerated tau accumulation over time. The Aβ+/SSD− group exhibited greater tau accumulation than the Aβ−/SSD− group, while no significant differences were observed between the Aβ− groups. Furthermore, the Aβ+/SSD+ group exhibited the most pronounced longitudinal increases in tau SUVRs within these regions compared with the other groups. Taken together, these findings suggest that the relationship between SSD and tau accumulation is evident primarily in the context of Aβ positivity, implying that amyloid pathology may modulate how depressive symptoms relate to tau deposition in non-demented older adults.

Depression is characterized by mood disturbances, impaired attention and concentration, and a diminished sense of self-worth. While depression and AD are generally considered distinct clinical entities, they share several common features, complicating the understanding of their interrelationship and making it difficult to differentiate between the two conditions when they co-occur. Depression and cognitive impairment share a bidirectional relationship, where midlife or late-life depression symptoms are related to a higher risk of subsequent MCI, and individuals with MCI are more prone to developing depression (Guo et al., 2023). The deposition of AD-related biomarkers, including Aβ and tau, has been linked to depression, further complicating the relationship between depression and AD. A systematic review of 15 cross-sectional studies has provided evidence for a potential link between amyloid pathology and MDD in older adults (Harrington et al., 2015). Moreover, SSD has been found to be associated with higher cerebrospinal fluid (CSF) amyloid levels and an 83% increased likelihood of developing AD in elderly adults without dementia (Xu et al., 2021). The relationship between SSD and Aβ pathology was bidirectional, with the effects of depressive symptoms on cognitive impairment and AD risk being partially mediated by Aβ pathology (Xu et al., 2021). In terms of tau pathology, a meta-analysis indicated that CSF total tau levels were similar in individuals with MDD and healthy controls. Cross-sectional neuroimaging and postmortem studies have reported associations between depressive symptoms and elevated cerebral tau burden (Gatchel et al., 2017; Babulal et al., 2020; Gonzales et al., 2021; Moriguchi et al., 2021; Tommasi et al., 2021), suggesting a potential contribution of depression to early tau pathology. Building on prior research, our results reveal significant interactions between SSD and Aβ status in relation to tau accumulation, as measured by tau PET. Specifically, we observed a significant interaction among Aβ status, time, and SSD in relation to tau accumulation in the Braak III/IV and Braak V/VI regions. These findings indicate that SSD is associated with faster tau accumulation primarily among Aβ-positive individuals, with no association observed in Aβ-negative participants. Together, these results suggest that amyloid pathology may modulate the relationship between depressive symptoms and tau deposition.

Several mechanisms may explain the interaction between depressive symptoms and Aβ pathology in tau accumulation in AD. First, depressive symptoms have been associated with impaired hippocampal neurogenesis (Kreisel et al., 2014), which may exacerbate neurodegenerative processes and accelerate the accumulation of toxic proteins in AD, including Aβ and tau. Second, chronic inflammation has been identified as a key mechanism linking depression to AD (Hayley et al., 2020). Recent studies have demonstrated that inflammatory alterations in the CSF closely parallel the burden of Aβ and tau (Cullen et al., 2021), while sustained activation of glial cells and inflammatory signaling pathways further exacerbates tau hyperphosphorylation and propagation (Chen and Yu, 2023). Third, depressive symptoms have been associated with dysregulation of the hypothalamic–pituitary–adrenal (HPA) axis, leading to chronically elevated glucocorticoid levels that may contribute to neurodegeneration (Galts et al., 2019). Moreover, sustained glucocorticoid exposure increases neuronal activity and Aβ release, thereby facilitating Aβ aggregation into plaques (Dong and Csernansky, 2009). In addition, experimental studies have further shown that stress-level glucocorticoid exposure increases Aβ production and promotes tau accumulation, suggesting that elevated glucocorticoids accelerate both Aβ and tau pathology (Green et al., 2006). These mechanisms collectively point to a complex interaction between depression, Aβ pathology, and tau accumulation, with implications for AD progression.

The relationship between depression and tau pathology may be bidirectional, with SSD potentially representing the downstream clinical phenotype of tauopathy. Several studies suggest that neurodegeneration may be a key cause of depression, disrupting circuits involved in emotional regulation. A significant correlation between tau levels and psychological symptoms of dementia was found in a study of memory clinic patients (Cotta Ramusino et al., 2021). Similarly, a study involving older adults demonstrated that elevated tau levels were associated with an increased likelihood of depression, with participants who had elevated tau being twice as likely to be depressed (Babulal et al., 2020). Moreover, elevated plasma total tau levels have been shown to be significantly associated with symptoms of depression, apathy, anxiety, worry, and sleep disturbances (Hall et al., 2021), and higher CSF tau levels have been associated with a greater risk of depression and apathy over time (Banning et al., 2021). These findings support the hypothesis that tau pathology may not only be a consequence of depression but may also contribute to the onset of depressive symptoms. Thus, future studies are needed to further investigate the bidirectional relationship between depressive symptoms and tau pathology.

Several limitations should be acknowledged when interpreting these findings. First, although all non-demented participants were included, the Aβ+/SSD+ group remained relatively small, which may limit the robustness and generalizability of group-specific results. Second, participants were drawn from the ADNI cohort, which predominantly includes well-educated, health-conscious volunteers, potentially limiting the representativeness of the sample. Third, the inclusion of both CN and MCI participants may have introduced residual heterogeneity. Although diagnostic status (CN vs. MCI) was included as a covariate in all statistical models, unmeasured diagnostic differences may still have influenced the results. Fourth, external validation was not feasible as other publicly available cohorts currently lack tau PET imaging data, limiting replication of tau-related findings. Fifth, the relatively short follow-up duration for longitudinal tau PET may have reduced sensitivity to detect slower or nonlinear trajectories of tau accumulation, potentially underestimating long-term effects. Finally, given the observational design, the reported associations among SSD, Aβ pathology, and tau accumulation should not be interpreted as causal. Future studies incorporating larger and more heterogeneous cohorts, longer longitudinal follow-up, and multimodal biomarker approaches will be essential to replicate and extend these findings.

In conclusion, this study demonstrates that SSD is associated with greater tau accumulation primarily in individuals with Aβ positivity, suggesting that amyloid pathology modulates the relationship between depressive symptoms and tau pathology during the early stages of AD. These findings indicate that SSD may serve as an early clinical marker of increased vulnerability to tau aggregation in the presence of Aβ pathology. Further longitudinal and mechanistic studies are warranted to clarify the underlying biological pathways and to determine whether early identification and management of SSD could help mitigate tau-related neurodegeneration.

## Data Availability

Publicly available datasets were analyzed in this study. This data can be found here: https://adni.loni.usc.edu.

## Footnotes

1
http://adni.loni.usc.edu

## Group member of the Alzheimer’s Disease Neuroimaging Initiative

Data used in the preparation of this article were obtained from the Alzheimer’s Disease Neuroimaging Initiative (ADNI) database (http://adni.loni.usc.edu). As such, the investigators within the ADNI contributed to the design and implementation of ADNI and/or provided data but did not participate in the analysis or writing of this report. A complete listing of ADNI investigators can be found at: https://adni.loni.usc.edu/wp-content/uploads/how_to_apply/ADNI_Acknowledgement_List.pdf.
