# Supplementary material for: Amyloid pathology modulates the relationship between subsyndromal symptomatic depression and tau accumulation in non-demented older adults
Source: Front Aging Neurosci. 2025 Dec 10;17:1679285. doi: 10.3389/fnagi.2025.1679285 (PMC12727920; doi:10.3389/fnagi.2025.1679285)
Supplement: Supplementary file 1 [file Table_1.docx]

**Amyloid Pathology Modulates the Relationship Between Subsyndromal Symptomatic Depression and Tau Accumulation in Non-Demented Older Adults**

**SUPPLEMENTAL MATERIAL**

Supplementary Table S1. Associations of Aβ status, SSD, and their interaction with baseline square-root transformed regional tau-PET SUVRs among non-demented participants (sensitivity analysis).

|  | **Braak I SUVR** | | |  | **Braak III/IV SUVR** | | |  | **Braak V/VI SUVR** | | |  | **Meta Temporal ROI SUVR** | | |
| --- | --- | --- | --- | --- | --- | --- | --- | --- | --- | --- | --- | --- | --- | --- | --- |
| Term | *β* | *95% CI* | *P* |  | *β* | *95% CI* | *P* |  | *β* | *95% CI* | *P* |  | *β* | *95% CI* | *P* |
| APOE ε4 | 0.25 | 0.06 ~ 0.44 | 0.01 |  | 0.16 | -0.04 ~ 0.36 | 0.109 |  | 0.08 | -0.12 ~ 0.29 | 0.417 |  | 0.17 | -0.03 ~ 0.37 | 0.088 |
| Age | -0.02 | -0.11 ~ 0.07 | 0.66 |  | -0.04 | -0.13 ~ 0.06 | 0.426 |  | -0.14 | -0.24 ~ -0.04 | 0.005 |  | -0.01 | -0.11 ~ 0.08 | 0.815 |
| Gender | 0.11 | -0.06 ~ 0.29 | 0.214 |  | 0.15 | -0.04 ~ 0.34 | 0.116 |  | 0.29 | 0.10 ~ 0.48 | 0.003 |  | 0.15 | -0.03 ~ 0.34 | 0.108 |
| Education | 0.1 | 0.01 ~ 0.19 | 0.023 |  | 0.05 | -0.04 ~ 0.14 | 0.288 |  | 0.08 | -0.02 ~ 0.17 | 0.104 |  | 0.03 | -0.06 ~ 0.12 | 0.522 |
| Diagnosis | 0.28 | 0.18 ~ 0.37 | <0.001 |  | 0.22 | 0.12 ~ 0.32 | <0.001 |  | 0.16 | 0.06 ~ 0.26 | 0.002 |  | 0.24 | 0.14 ~ 0.34 | <0.001 |
| SSD | -0.23 | -0.45 ~ -0.01 | 0.039 |  | -0.19 | -0.42 ~ 0.05 | 0.117 |  | -0.12 | -0.35 ~ 0.12 | 0.332 |  | -0.17 | -0.40 ~ 0.05 | 0.135 |
| Aβ | 0.39 | 0.13 ~ 0.66 | 0.004 |  | 0.43 | 0.15 ~ 0.71 | 0.003 |  | 0.46 | 0.17 ~ 0.75 | 0.002 |  | 0.43 | 0.15 ~ 0.71 | 0.002 |
| SSD+ × Aβ+ | 0.64 | 0.29 ~ 0.98 | **<0.001** |  | 0.51 | 0.14 ~ 0.87 | **0.006** |  | 0.45 | 0.08 ~ 0.82 | **0.017** |  | 0.51 | 0.16 ~ 0.87 | **0.005** |

Abbreviations: All regional tau-PET SUVRs were square-root–transformed. Aβ+, amyloid-β–positive; APOE, apolipoprotein E; SSD, subsyndromal symptomatic depression; SSD+, presence of subsyndromal depressive symptoms, defined as a Geriatric Depression Scale (GDS-15) score of 1–5; SUVR, standardized uptake value ratio; CI, confidence interval. Linear regression models were adjusted for age, gender, years of education, diagnostic status (CN vs. MCI), and APOE ε4 carrier status. β values represent the estimated effect size for each predictor on square-root–transformed regional tau-PET SUVRs. *p* values in bold indicate statistical significance (*p* < 0.05).

Supplementary Table S2. Longitudinal linear mixed-effects models for changes in square-root transformed regional tau-PET SUVRs among non-demented participants (sensitivity analysis).

|  | **Braak I SUVR** | |  |  | **Braak III/IV SUVR** | |  |  | **Braak V/VI SUVR** | |  |  | **Meta Temporal ROI SUVR** | | |
| --- | --- | --- | --- | --- | --- | --- | --- | --- | --- | --- | --- | --- | --- | --- | --- |
| Term | *Estimate* | *SE* | *P* |  | *Estimate* | *SE* | *P* |  | *Estimate* | *SE* | *P* |  | *Estimate* | *SE* | *P* |
| Age X time | 0.000 | 0.000 | 0.286 |  | 0.000 | 0.000 | 0.725 |  | 0.000 | 0.000 | 0.680 |  | 0.000 | 0.000 | 0.704 |
| APOE ε4 X time | 0.000 | 0.005 | 0.984 |  | 0.003 | 0.004 | 0.435 |  | 0.003 | 0.003 | 0.292 |  | 0.004 | 0.004 | 0.360 |
| Diagnosis X time | -0.003 | 0.005 | 0.473 |  | 0.007 | 0.004 | 0.056 |  | 0.008 | 0.003 | 0.018 |  | 0.008 | 0.004 | 0.040 |
| Education X time | 0.001 | 0.001 | 0.317 |  | 0.000 | 0.001 | 0.871 |  | 0.000 | 0.001 | 0.585 |  | 0.000 | 0.001 | 0.681 |
| Gender X time | 0.004 | 0.005 | 0.448 |  | 0.000 | 0.004 | 0.891 |  | 0.001 | 0.003 | 0.710 |  | 0.000 | 0.004 | 0.991 |
| Aβ X time | 0.004 | 0.008 | 0.634 |  | 0.003 | 0.006 | 0.575 |  | -0.001 | 0.005 | 0.898 |  | 0.006 | 0.007 | 0.381 |
| SSD X time | -0.006 | 0.007 | 0.401 |  | -0.010 | 0.006 | 0.062 |  | -0.011 | 0.005 | 0.029 |  | -0.009 | 0.006 | 0.165 |
| Aβ X SSD X time | 0.013 | 0.009 | 0.171 |  | 0.013 | 0.007 | 0.063 |  | 0.013 | 0.006 | **0.046** |  | 0.012 | 0.008 | 0.134 |

Abbreviations: All regional tau-PET SUVRs were square-root–transformed. Aβ, amyloid-β; SSD, subsyndromal symptomatic depression; APOE, apolipoprotein E; SUVR, standardized uptake value ratio. Linear mixed-effects models were used to evaluate longitudinal changes in tau-PET SUVRs across regions of interest (Braak I, Braak III/IV, Braak V/VI, and meta-temporal). First, changes related to Aβ status and SSD were assessed in the same model; then, the interaction between Aβ status and SSD with time (Aβ × SSD × time) was added. Main effects of independent variables are included in each model (estimates not shown). Estimates are unstandardized and reflect the yearly change in each square-root–transformed regional tau-PET SUVR. *p* values in bold indicate statistical significance ( *p* < 0.05).

Supplementary Table S3. Pairwise comparisons of longitudinal tau accumulation (square-root–transformed) across Aβ/SSD groups among non-demented participants (sensitivity analysis)

|  | Braak III/IV SUVR | |  |  | Braak V/VI SUVR | |  |
| --- | --- | --- | --- | --- | --- | --- | --- |
| Contrast | *Estimate* | *SE* | *P* |  | *Estimate* | *SE* | *P* |
| (Aβ−/SSD− X time) vs (Aβ−/SSD+ X time) | 0.017 | 0.009 | 0.057 |  | 0.010 | 0.007 | 0.153 |
| (Aβ−/SSD− X time) vs (Aβ+/SSD− X time) | -0.032 | 0.011 | **0.004** |  | -0.026 | 0.009 | **0.003** |
| (Aβ−/SSD− X time) vs (Aβ+/SSD+ X time) | -0.055 | 0.010 | **<0.001** |  | -0.045 | 0.008 | **<0.001** |
| (Aβ−/SSD+ X time) vs (Aβ+/SSD− X time) | -0.049 | 0.010 | **<0.001** |  | -0.036 | 0.008 | **<0.001** |
| (Aβ−/SSD+ X time) vs (Aβ+/SSD+ X time) | -0.072 | 0.010 | **<0.001** |  | -0.055 | 0.008 | **<0.001** |
| (Aβ+/SSD− X time) vs (Aβ+/SSD+ X time) | -0.023 | 0.011 | **0.041** |  | -0.019 | 0.009 | **0.039** |

Abbreviations: All regional tau-PET SUVRs were square-root–transformed. Aβ−, amyloid-β–negative; Aβ+, amyloid-β–positive; SSD, subsyndromal symptomatic depression; SSD+, presence of subsyndromal depressive symptoms, defined as a Geriatric Depression Scale (GDS) score of 1–5; SSD−, absence of depressive symptoms (GDS = 0); SUVR, standardized uptake value ratio. Estimates represent unstandardized regression coefficients derived from linear mixed-effects models and indicate the rate of annual change in square-root–transformed regional tau-PET SUVRs. *p* values in bold indicate statistical significance after FDR correction (adjusted *p* < 0.05).
